# Crowded catalyst, better catalyst

**DOI:** 10.1093/nsr/nwab141

**Published:** 2021-08-09

**Authors:** Matteo Monai, Bert M Weckhuysen

**Affiliations:** Inorganic Chemistry and Catalysis, Debye Institute for Nanomaterials Science, Utrecht University, The Netherlands; Inorganic Chemistry and Catalysis, Debye Institute for Nanomaterials Science, Utrecht University, The Netherlands

At the time of writing (during the pandemic), thinking about crowded spaces may make us uncomfortable, or melancholic at times. Some of us may even shy away from crowds by nature. But in their recent research paper in *Nature*, Ding Ma *et al.* show us that crowds can be good after all, at least at the nanoscopic level [1].

**Figure 1.**
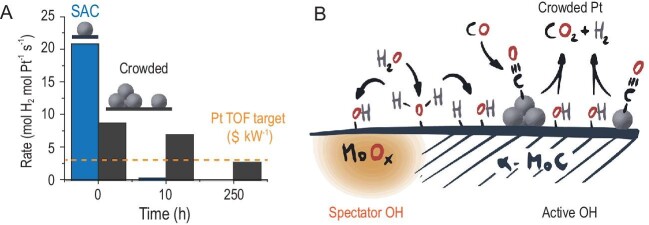
(A) Catalytic rate in the water gas shift (WGS) reaction observed for the Pt/α-MoC catalysts reported by Ding Ma *et al.* (see ref. [1]), for a single atom Pt catalyst and a crowded Pt catalyst, composed of single atoms and small nanoclusters. The rate is compared to turnover frequency (TOF) targets for WGS reaction Pt catalysts in fuel cells. (B) The proposed mechanism of stabilization of crowded Pt catalysts, involving rapid consumption of OH species, which otherwise lead to catalyst deactivation by formation of MoO_x_.

Bringing Pt single atoms and small Pt nanoclusters closer together on a molybdenum carbide (α-MoC) support, the authors produced a more stable water gas shift (WGS) catalyst, which showed activity at unprecedently low temperatures (equilibrium was reached at 100°C and high space velocity). This is an exciting step towards more sustainable hydrogen-fueled transportation fuel cells, as these Pt/α-MoC catalysts are the first to meet stringent targets set by the US Department of Energy (Fig. 1A). But what is so special about them?

The key lies in stabilizing the α-MoC support against oxidation by OH groups formed during the WGS reaction by water activation (Fig. 1B). Crowding the support with Pt species makes the removal of such OH groups favorable, and thus prevents the formation of MoO_x_ on the catalyst surface. Using near ambient pressure X-ray photoelectron spectroscopy (NAP-XPS) and transient kinetic analysis (TKA) the authors showed that OH species far from the Pt species act as spectators during the reaction, while OH groups adjacent to Pt could be eliminated by CO adsorbed on Pt species, freeing up the α-MoC surface site for further water dissociation. Crowding Pt also leads to high mass-specific catalytic activity, because of the higher loading used per gram of material with respect to traditional single atom catalysts (SACs).

Such findings highlight the importance of interparticle distance in catalysis, and resonate with previous reports of particle proximity effects in electrocatalysis, where OH coverage and activity in the oxygen reduction reaction over Pt-based catalysts were modulated by an enhancement of the electric potential in the electric double layer in closely packed nanoparticle ensembles [2]. Controlling interparticle distance with self-assembly approaches may be regarded as a general strategy to improve catalysis, provided particles can be stabilized against aggregation and growth. On the other hand, such proximity effects introduce yet another level of complexity in understanding structure–performance relationships in catalysis at different scales. New *operando* methodologies that allow the study of single particles in large ensembles (e.g. model catalyst beds) hold promise for disentangling different effects and exploring catalyst landscapes under a new light [3].

Another take-home message of the paper is that support engineering is equally as important as the more popular active phase design. Controlling the defects, facet exposures and chemical phase distribution of supports has been shown to lead to game-changing performances even on century-old active phases [4]. Surface-sensitive characterization techniques are of the essence here, to study subtle changes in surface chemistry in bulky supports, which can make up to 99 wt% of a catalyst. In the case of this paper, oxidation of α-MoC in less-crowded catalysts could only be evidenced by surface-sensitive XPS, and was not detected by bulk X-ray diffraction (XRD). Development of *in situ* and *operando* XPS techniques that can completely overcome the pressure gap is underway, and we believe it will lead to cornerstone advancements in understanding metal-support interactions in catalysis [5].

While Ding Ma's catalysts are promising candidates for low-temperature and less-energy-intensive hydrogen purification, it remains to be seen how such crowded Pt species will respond to the highly variable conditions of typical stationary fuel cell systems, in which numerous redox cycles and rapid start-ups and shut-downs could cause deactivation via restructuring and nanoparticles aggregation. Furthermore, extending such an approach to base metals (e.g. the commercially used Cu-Zn- and Fe-Cr-based catalysts) would be an interesting strategy with regard to possibly finding alternatives to the much-less-sustainable Pt. We certainly hope that, for once, we may achieve great things by ‘following the crowd’.

***Conflict of interest statement*.** None declared.
